# A signature of 18 immune‐related gene pairs to predict the prognosis of pancreatic cancer patients

**DOI:** 10.1002/iid3.363

**Published:** 2020-10-31

**Authors:** Fanqin Bu, Han Nie, Xiaojian Zhu, Ting Wu, Kang Lin, Jiefeng Zhao, Jun Huang

**Affiliations:** ^1^ Department of Gastrointestinal Surgery Second Affiliated Hospital of Nanchang University Nanchang Jiangxi China; ^2^ Department of Vascular Surgery Second Affiliated Hospital of Nanchang University Nanchang Jiangxi China; ^3^ Zhongshan School of Medicine Research Center of the Seventh Affiliated Hospital of Sun Yat‐Sen University Shenzhen China; ^4^ Infection Department of Guixi Traditional Chinese Medicine Hospital Guixi Jiangxi China

**Keywords:** immune‐related gene pair, pancreatic cancer, prognostic signature

## Abstract

Pancreatic cancer is one of the most lethal malignancies. With the promising prospects conveyed by immunotherapy in cancers, we aimed to construct an immune‐related gene pairs (IRGPs) signature to predict the prognosis of pancreatic cancer patients. We downloaded clinical and transcriptional data of pancreatic cancer patients from The Cancer Genome Atlas data set as the training group and GSE57495 data set as the verification group. We filtered immune‐related transcriptional data by IMMPORT. With the assistance of lasso penalized Cox regression, we constructed our prognostic IRGPs signature and divided all samples into high‐/low‐risk groups by receiver operating characteristic curve for further comparisons. The comparisons between high‐ and low‐risk groups including survival rate, multivariate, and univariate Cox proportional‐hazards analysis, infiltration of immune cells, and Gene Set Enrichment Analysis (GSEA). Gene Ontology (GO), Kyoto Encyclopedia of Genes and Genomes (KEGG) are facilitated to analyze the proceedings in which our IRGPs signature may involve in. The results revealed that 18 IRGPs were defined as our prognostic signature. The prognostic value of this IRGPs signature was verified from the GSE57495 data set. We further demonstrated the independent prognostic value of this IRGPs signature. The contents of six immune cells between high‐/low‐risk groups were different, which was associated with the progression of diverse cancers. Results from GO, KEGG, and GSEA revealed that this IRGPs signature was involved in extracellular space, immune response, cancer pathways, cation channel, and gated channel activities. Evidently, this IRGPs signature will provide remarkable value for the therapy of pancreatic cancer patients.

## Graphical abstract

In our study, we aimed to construct an immune‐related gene pairs (IRGPs) signature to predict the prognosis of pancreatic cancer patients. We will verify the validity of our model by receiver operating characteristic curve, prognostic analysis, multivariate and univariate Cox proportional‐hazards analysis, and infiltration of different immune cells. Unlike traditional model, the pairwise comparison of each IRGP and the calculation of the score were absolutely based on the genetic expressions in the same patient thus it is not a necessity in our IRGPs signature to standardize the genetic expression profiles from different sequencing platforms.

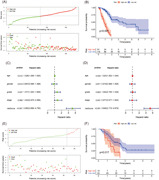

## 1 INTRODUCTION

Pancreatic cancer (PC) is considered to be one of the most lethal malignant tumors, and its global incidence is expected to rise to approximately 420,000 cases by 2020, unfortunately, the 5‐year survival rate for PC patients is less than 10%.^1^, ^2^ Currently, an increasing number of people are confronting obesity and diabetes diseases, which are tightly correlated with the development of PC.^2^ The anatomical location of the pancreas is deep in the abdomen thus leads to the difficulty of diagnosis at early stages. Unfortunately, surgical treatment for patients with advanced PC is not feasible, therefore, other treatment procedures including immunotherapy, chemotherapy, and radiotherapy were urged to be applied to PC.^3^

Recently, dysregulation of the immune system has been reported to correlate with the development of malignant tumors. Therefore, immunotherapy has become a crucial strategy for the treatment of various of cancers.^4^, ^5^, ^6^ Previous studies also implied the potential validity to apply immunotherapy into PC patients. Peripheral blood analysis revealed that the contents of CD8^+^ T cells were significantly lower in PC patients than in healthy controls and higher infiltration of CD4^+^/CD8^+^ T cells corresponds to better survival in PC patients.^7^, ^8^ Th1 cells and Th2 cells are originated from the differentiation of Naive CD4^+^ T cells, the diversion from Th1 to Th2 cells associated with poor survival in PC patients.^9^ CD226 and CD96 were reported to regulate the functions of natural killer (NK) cells, the contents of CD226^+^ and CD96^+^ NK cells were lower in PC patients comparing to healthy groups. Moreover, the reduction of CD226^+^ and CD96^+^ NK cells is correlate with tumor histological grade and lymph node metastasis, and the decreased percentages of CD226^+^ and CD96^+^ NK cells could cause tumor immune escape in PC patients.^10^ Above all, it is considerable to apply immunotherapy into PC patients.

To date, none has applied immune‐related genes (IRGs) into the therapy of PC patients. To provide an original method into the treatment of PC, we performed bioinformatic methods to construct a prognostic IRGPs signature. Data of PC patients were downloaded from The Cancer Genome Atlas (TCGA) and GEO database (GSE57495) and we further employed IMMPORT to filter the IRGs of our transcriptional data. Ultimately, we constructed and validated the prognostic value of our IRG pairs (IRGPs) signature. Taken together, this study will facilitate the application of immunotherapy into PC.

## 2 METHODS

### 2.1 Summary

This is a study based on TCGA data and GEO data to perform construction of immune‐related prognostic signature. The TCGA group was deemed as the training group, and GSE57495 data set was employed for the verification group. RNA‐seq expression data and clinical data of PC patients of the two data sets were downloaded. With the combination of Immport database, we kept the immune‐related transcriptional expression data and further utilize the data with survival time to dig out prognostic‐relevant IRGPs. A well‐balanced model that contains 18 IRGPs was constructed by lasso penalized Cox regression. In the meanwhile, we divided all samples into high‐/low‐risk groups by the optimal cut‐off in ROC curve analysis. This IRGPs model was validation by the overall survival difference in the verification group. We also performed univariate/multivariate Cox proportional‐hazards analysis in the training group to identify our model as an independent prognostic factor. We further compared the infiltration of immune cells between high‐/low‐risk groups and performed Gene Set Enrichment Analysis (GSEA) of the two groups. Gene Ontology (GO), Kyoto Encyclopedia of Genes and Genomes (KEGG) analysis were performed on DAVID database. Expression profile, clinical relevance, and mutational analysis of the 18 IRGPs in PC was performed on GEPIA2 and cBioportal platform.

### 2.2 Sources of PC patients

TCGA (https://portal.gdc.cancer.gov) PC samples, a total of 177 samples that both contain RNA‐seq expression data and clinical data were selected for the test group. GEO (http://www.ncbi.nlm.nih.gov/geo/) PC samples, GSE57495 (in GPL15048) contained 63 PC samples with survival time, are selected as the verification group to verify the validation of our model. All samples are available in our study.

### 2.3 Data preprocessing

When patients appear in the database more than once, we average their expression profile data. When a target gene matches multiple probes, we average the probes to represent the expression level of the gene.

### 2.4 IRGs extraction

Immport (https://immport.niaid.nih.gov) was one of the largest open repositories of human immunological data.^11^ We downloaded a list of 2498 IRGs from Immport. Then we discard the immune‐unrelated genes from our transcriptional data.

### 2.5 Construction of the IRGPs signature

Each IRG was paired with each other and each IRGP (IRG pair) has a specific score. Detailly, in the pairwise comparison by R, the output is 1 if the expression of the first immune gene in a specific sample is more than the following one and 0 for the reversed order. Subsequently, we deleted the IRGPs if the score of which were 0 or 1 in more than 80% of the samples, the remaining IRGPs were deemed as initial candidate IRGPs. We performed log‐rank test in our training group to obtain prognostic‐related IRGPs (*p* < .0001), moreover, we performed lasso penalized Cox regression (iteration = 1000) to obtain a well‐balanced prognostic model by R (glmnet package). Ultimately, the most stable model which contains 18 IRGPs was deemed as our final IRGPs signature. To classify patients into high‐/low‐risk groups, we performed time‐dependent receiver operating characteristic (ROC) curve analysis at 1 year in the training group for overall survival and obtained optimal cutoff. Patients with higher risk score than the optimal cutoff will be counted in high‐risk group, patients with lower risk score will be counted in low‐risk group.

### 2.6 Validation of the IRGPs signature

We employed R (survival package) to obtain the Kaplan–Meier curve for comparing the overall survival difference between high‐/low‐risk groups in TCGA and GSE57495 PC samples. Moreover, to validate our model to be an independent prognostic factor, we performed univariate and multivariate Cox proportional‐hazards analysis for the training group to assess our prognostic model with other clinical factors.

### 2.7 Comparison of the infiltration of immune cells between high‐/low‐risk groups

CIBERSORT (http://cibersort.stanford.edu/) has been widely used for analyzing the cellular composition of a tissue from its gene expression profile, especially for analyzing the composition of immune cells in tumors.^12^ We compared the infiltration of 22 immune cells between high‐/low‐risk groups in TCGA PC samples. Results of *p* < .05 was regarded as statistically significant.

### 2.8 GSEA

To compare the differential enrichment of gene sets between the high‐/low‐risk groups, we employed R (Bioconductor package fgsea) to perform GSEA and uncover potential biological mechanisms that our prognostic model may involve in. GO gene sets (c5.all.v7.0.symbols.gmt) in GSEA database were downloaded for our study. Gene sets with the results of FDR value < 0.05 were deemed to be statistically valuable.

### 2.9 GO and KEGG analysis

DAVID database is used for analyze the functions of genomic statistics and further classify the data.^13^ We employed DAVID to perform functional enrichment analysis including biological process, molecular function, cell component, and KEGG pathway of the genes in the IRGPs signature.

### 2.10 GEPIA2

GEPIA2 (http://gepia2.cancer-pku.cn/) is a multifunctional molecular analysis platform based on TCGA data and GETx data which contains 179 PC samples and 171 normal samples altogether.^14^ It was employed to comparing the expression profile of each IRGP between PC and adjacent normal tissues. The results of *p* value were generated by Student's *t* test. Differential expression of an IRGP in the two sets of tissues was said to occur only if the results of *p* < .05 and |Log_2_FC| > 1. Moreover, we investigated the relationship between the expression of each IRGP and the PC stage. It was said an IRGP was correlated with tumor stage only when both genes in an IRGP are related to the stage of PC (*p* < .05).

### 2.11 cBioportal

cBioportal (https://www.cbioportal.org/) is an online database used for exploring, visualizing, and analyzing different cancer genomics data.^15^ This tool was employed to analyze genetic alterations including alteration rate and detailed categories of genetic alterations of each IRGP in PC.

### 2.12 Statistical analysis

We employed R (version 3.6.1) to execute all proceedings. The packages in R were listed in each proceeding (*p < .05, **p < .01, and ***p < .001).

## 3 RESULTS

### 3.1 Summary

We constructed an IRGPs signature to predict the prognosis of PC patients. This signature was correlated with the infiltration of immune cells and cancer‐related pathways.

### 3.2 The construction of IRGPs signature

**Figure 1:**
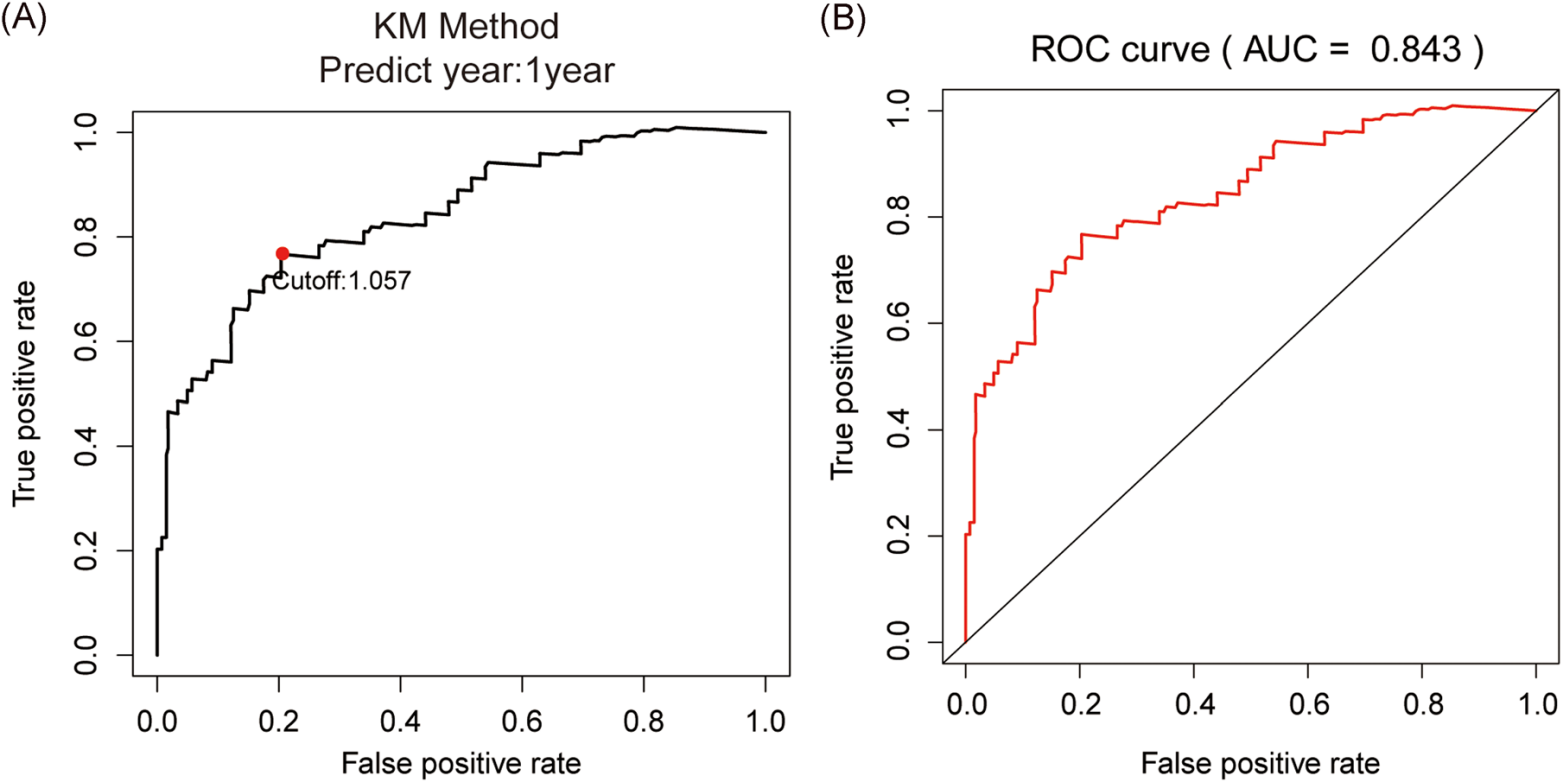
Construction of the immune‐related gene pairs (IRGPs) signature. (A) Time‐dependent receiver operating characteristic (ROC) curve analysis (1 year) for IRGPs signature in the training group, the optimal cutoff is 1.057 to classify patients into high‐/low‐risk groups. (B) Area under receiver operating characteristic curve (AUC) is 0.843

**Figure 2:**
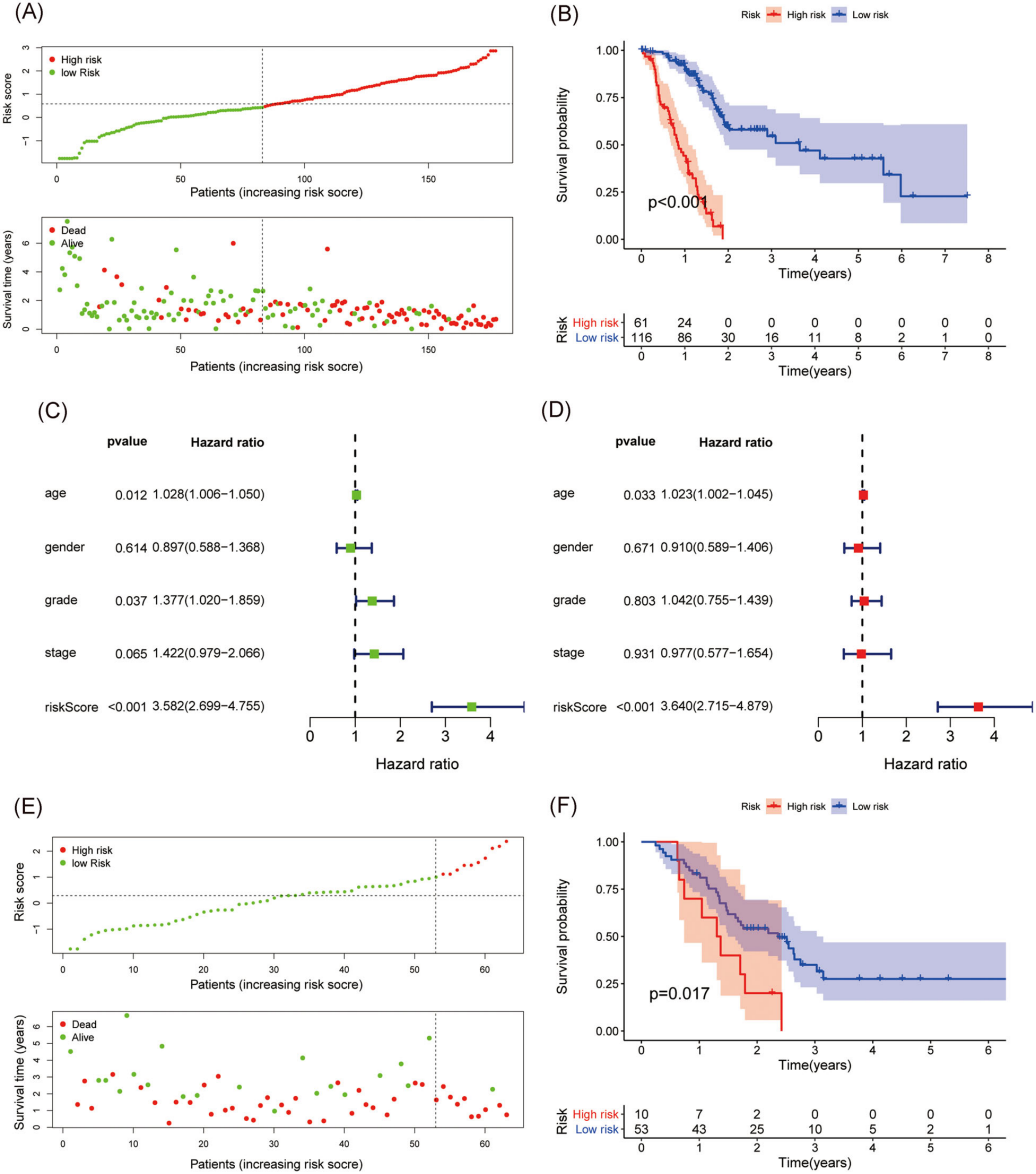
Validation of the immune‐related gene pairs (IRGPs) signature. (A) Divide the training‐group patients into high‐/low‐risk groups. (B) The comparison of overall survival rate between high‐/low‐risk groups in patients from The Cancer Genome Atlas (TCGA) data set. (C) Univariate Cox proportional‐hazards analysis of the risk factors in the training group. (D) Multivariate Cox proportional‐hazards analysis of the risk factors in the training group. (E) Divide the validation‐group patients into high‐/low‐risk groups. (F) The comparison of overall survival rate between high‐/low‐risk groups in patients from GEO (GSE57495) data set

We selected TCGA PC samples (*n* = 177) with transcriptional data and clinical data as our test group and data from GEO PC samples GSE57495 (*n* = 63) as the verification group. The clinical data of these two data sets are shown in Table 1. 2498 IRGs from IMMPORT database are downloaded for our data screening, and we ultimately keep IRGs in our transcriptional data. Moreover, we paired our IRG with each other, and we deleted the IRGPs if the score of which were 0 or 1 in more than 80% of the samples, in that case, 26524 IRGPs were remained. We performed a log‐rank test in our training group and further obtaining 33 prognostic IRGPs (*p* < .0001). Subsequently, we performed lasso penalized Cox regression (iteration = 1000) to define an index of each IRGPs, the risk score of each patient was calculated by these indexes. We further obtained a well‐balanced prognostic model, the 18 IRGPs (Table 2), were selected to construct the signature. To divide all the samples into high‐/low‐risk groups, we performed time‐dependent ROC curve analysis at 1 year in the training group for overall survival and we obtained the optimal cutoff of 1.057 (Figure 1A). Patients with higher risk score than 1.057 will be counted in high‐risk group, patients with lower risk score than 1.057 will be counted in low‐risk group. The classification of high‐/low‐risk groups of patients in TCGA data set was shown in Figure 2A, THE classification of high‐/low‐risk groups of patients in GSE57495 data set was shown in Figure 2E. The area under receiver operating characteristic curve (AUC) of 1‐year survival rate was 0.843, which demonstrated the validity of our prognostic signature (Figure 1B).

**Table 1:** Clinical data of The Cancer Genome Atlas (TCGA) data set and GSE57495 data set

	TCGA	GSE57495
Age (year)		
<60	54 (30.5%)	–
≥60	123 (69.5%)	–
Gender		
Female	79 (44.6%)	–
Male	98 (55.4%)	–
Survival status		
Alive	84 (47.5%)	21 (33.3%)
Dead	93 (52.5%)	42 (66.7%)
Grade		
Grade 1	28 (15.8%)	–
Grade 2	96 (54.2%)	–
Grade 3	51 (28.8%)	–
Grade 4	2 (1.1%)	–
Stage		
Stage I	19 (10.7%)	13 (20.6%)
Stage II	149 (84.2%)	50 (79.4%)
Stage III	4 (2.3%)	0
Stage IV	5 (2.8%)	0

**Table 2:** IRGPs signature

IRG1	Full name	IRG2	Full name	Coefficient
CD1C	CD1c Molecule	MUC5AC	Mucin 5AC, oligomeric mucus/gel‐forming	−0.036
CD1D	CD1d Molecule	DKK1	Dickkopf WNT signaling pathway inhibitor 1	−0.209
ICAM1	Intercellular cell adhesion molecule‐1	MET	Mesenchymal‐epithelial transition factor	−0.023
ERAP2	Endoplasmic reticulum aminopeptidase 2	SSTR1	Somatostatin receptor 1	0.739
CXCL9	Chemokine (C‐X‐C motif) ligand 9	APLNR	Apelin receptor	0.365
CXCL11	Chemokine (C‐X‐C motif) ligand 11	CD79A	CD79a molecule	0.119
CXCL11	Chemokine (C‐X‐C motif) ligand 11	PIK3R5	Phosphoinositide‐3‐kinase regulatory subunit 5	0.135
CXCL11	Chemokine (C‐X‐C motif) ligand 11	PRKCB	Protein kinase C β	0.165
CXCL11	Chemokine (C‐X‐C motif) ligand 11	ZAP70	Zeta chain of T‐cell receptor associated Protein kinase 70	0.341
PLAU	Urokinase‐type plasminogen activator	ZYX	Zyxin	0.018
IRF3	Interferon regulatory factor 3	MET	Mesenchymal‐epithelial transition factor	−0.454
IL1A	Interleukin 1α	CCL23	C‐C motif chemokine ligand 23	0.015
OAS1	2′,5′‐oligoadenylate synthetase 1	AGT	Angiotensinogen	0.260
AGER	Advanced glycosylation end‐product specific receptor	IL20RB	Interleukin 20 receptor subunit β	−0.300
PPARG	Peroxisome proliferator activated receptor γ	FGR	FGR proto‐oncogene, Src family tyrosine kinase	0.442
CHGA	Chromogranin A	IL22RA1	Interleukin 22 receptor subunit‐α 1	−0.571
EREG	Epiregulin	RARB	Retinoic acid receptor‐β	0.260
GMFB	Glia maturation factor β	TGFA	Transforming growth factor‐α	−0.173

### 3.3 Validation of the IRGPs signature

The results both from the test group (*p* < .001) and verification group (*p *= .017) revealed that PC patients in the high‐risk group have poor overall survival rate (Figures 2B and 2F). We further performed univariate and multivariate cox proportional‐hazards analysis for the test group. Results of univariate Cox proportional‐hazards analysis revealed that our IRGPs signature (*p* < .001, hazard ratio [HR]: 3.582, 95% confidence interval [CI]: 2.699–4.755), age (*p *= .012, HR: 1.028, 95% CI: 1.006–1.050) and tumor grade (*p *= 0.037, HR: 1.377, 95% CI: 1.020–1.859) were correlated with the prognosis of PC patients (Figure 2C). Results of multivariate Cox proportional‐hazards analysis demonstrated the independent prognostic value of our IRGPs signature (*p* < .001, HR: 3.640, 95% CI: 2.715–4.879) (Figure 2D).

### 3.4 Differences of the infiltration of immune cells between high‐/low‐risk groups

**Figure 3:**
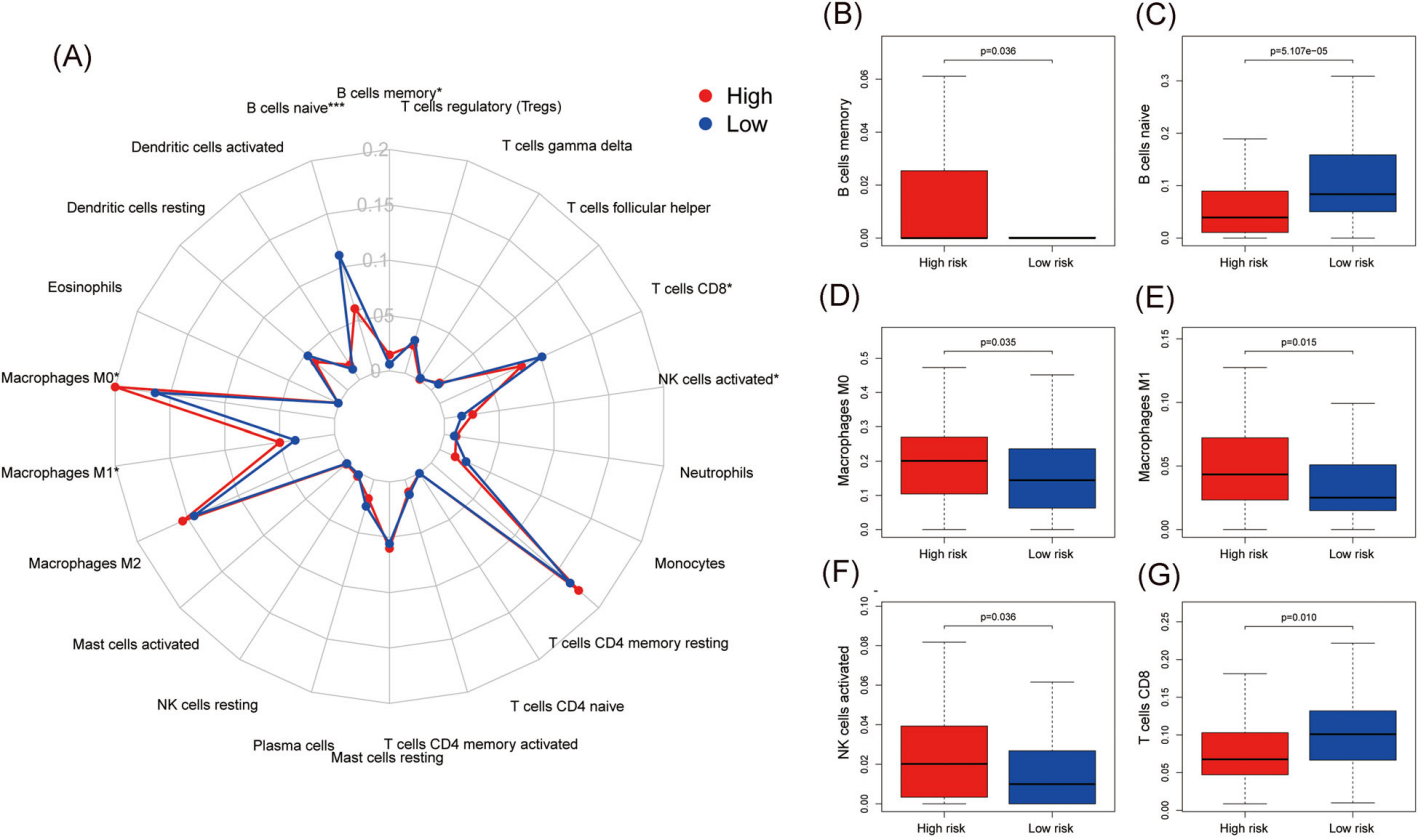
Correlation between the immune‐related gene pairs (IRGPs) model with infiltration of immune cells. (A) Summarize the difference of infiltration of immune cells between high‐/low‐risk groups (*p < .05, **p < .01, and ***p < .001). (B) The content of B cells memory (*p* = .036) was higher in the high‐risk group. (C) The content of B cells naïve (*p* < .001) was lower in the high‐risk group. (D) The content of macrophages M0 (*p* = .035) was higher in the high‐risk group. (E) The content of macrophages M1 (*p* = .015) was higher in the high‐risk group. (F) The content of natural killer cells activated (*p* = .036) was higher in the high‐risk group. (G) The content of T cells CD8 (*p* = .010) was lower in the high‐risk group

Infiltration of immune cells was correlated with cancer development and prognosis. We employed CIBERSORT to compare the differential contents of 22 immune cells between the high‐/low‐risk groups. Among the 22 immune cells, the contents of six immune cells were different between the two groups (Figure 3A). The contents of B cells memory (*p* = .036) (Figure 3B), Macrophages M0 (*p* = .035) (Figure 3D), Macrophages M1 (*p* = .015) (Figure 3E) and NK cells activated (*p* = .036) (Figure 3F) were higher in the high‐risk group. The contents of B cells naïve (*p* < .001) (Figure 3C) and T cells CD8 (*p* = .010) (Figure 3G) were lower in the high‐risk group.

### 3.5 GSEA

**Figure 4:**
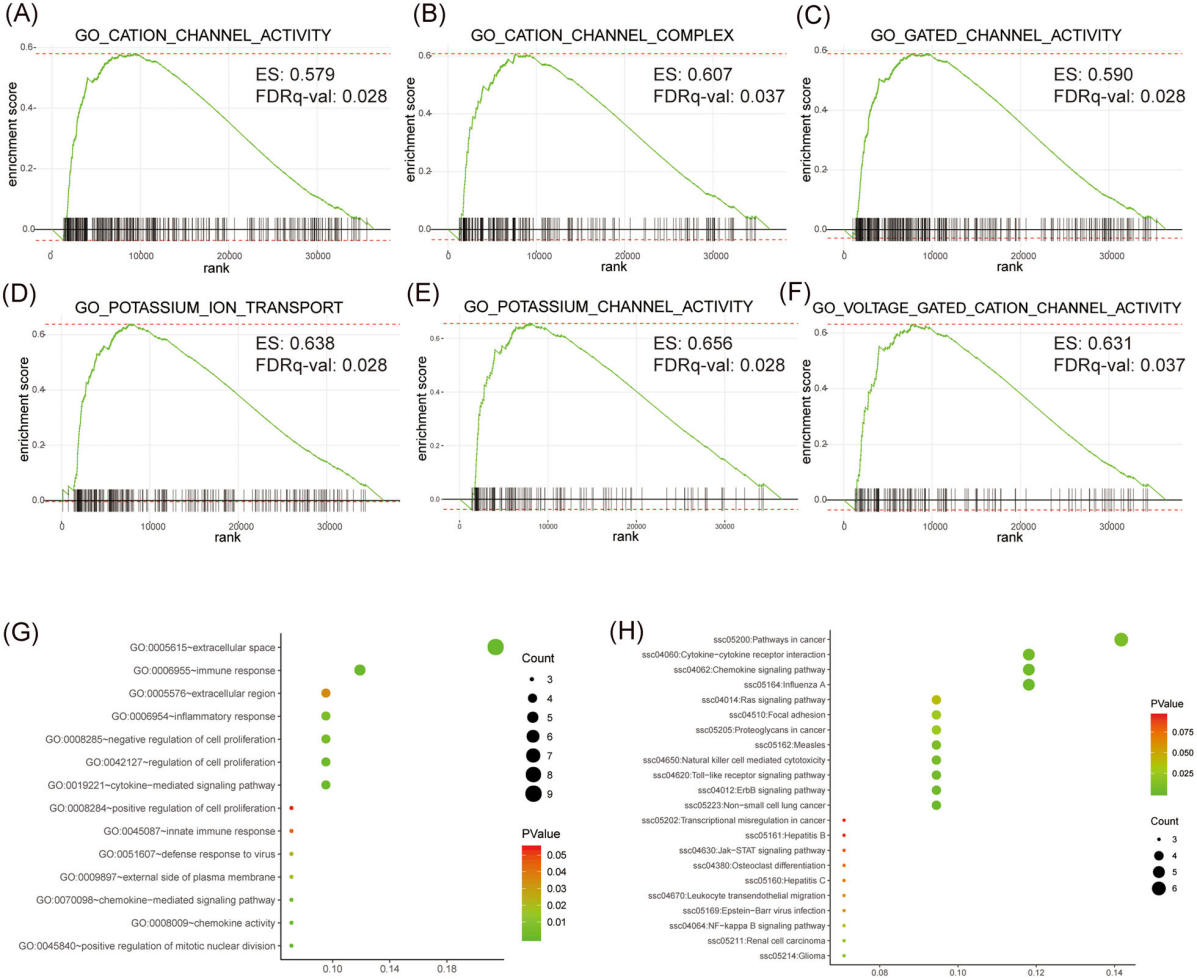
Gene Ontology (GO), Kyoto Encyclopedia of Genes and Genomes (KEGG), and Gene Set Enrichment Analysis (GSEA) of the immune‐related gene pairs (IRGPs) model. (A–F) GSEA analysis between the high‐/low‐risk groups. The results revealed six gene sets that the patients in the high‐risk group may involve in (results of FDR < 0.05 were listed). (G) GO analysis including biological process, molecular function and cell component of the genes in the IRGPs signature. (H) KEGG analysis predicted the biological pathways which our IRGPs signature may involve in

The intention of GSEA was to infer the potential biological mechanisms which the patients in the high‐risk group may involve in. The results of GSEA revealed that CATION_CHANNEL_ACTIVITY (Enrichment score: 0.579, FDR value = 0.028) (Figure 4A), CATION_CHANNEL_COMPLEX (Enrichment score: 0.607, FDR value = 0.037) (Figure 4B), GATED_CHANNEL_ACTIVITY (Enrichment score: 0.590, FDR value = 0.028) (Figure 4C), POTASSIUM_ION_TRANSPORT (Enrichment score: 0.638, FDR value = 0.028) (Figure 4D), POTASSIUM_CHANNEL_ACTIVITY (Enrichment score: 0.656, FDR value = 0.028) (Figure 4E) and VOLTAGE_GATED_CATION_CHANNEL_ACTIVITY (Enrichment score: 0.631, FDR value = 0.037) (Figure 4F) were significantly altered between high‐ and low‐risk groups.

### 3.6 GO and KEGG

GO analysis revealed that the genes in our IRGPs signature mainly enriched in extracellular space and immune response (Figure 4G). KEGG analysis revealed that these genes mainly involved in cancer pathways (Figure 4H).

### 3.7 Expression profile of each IRGP and the correlation with clinical parameters

**Figure 5:**
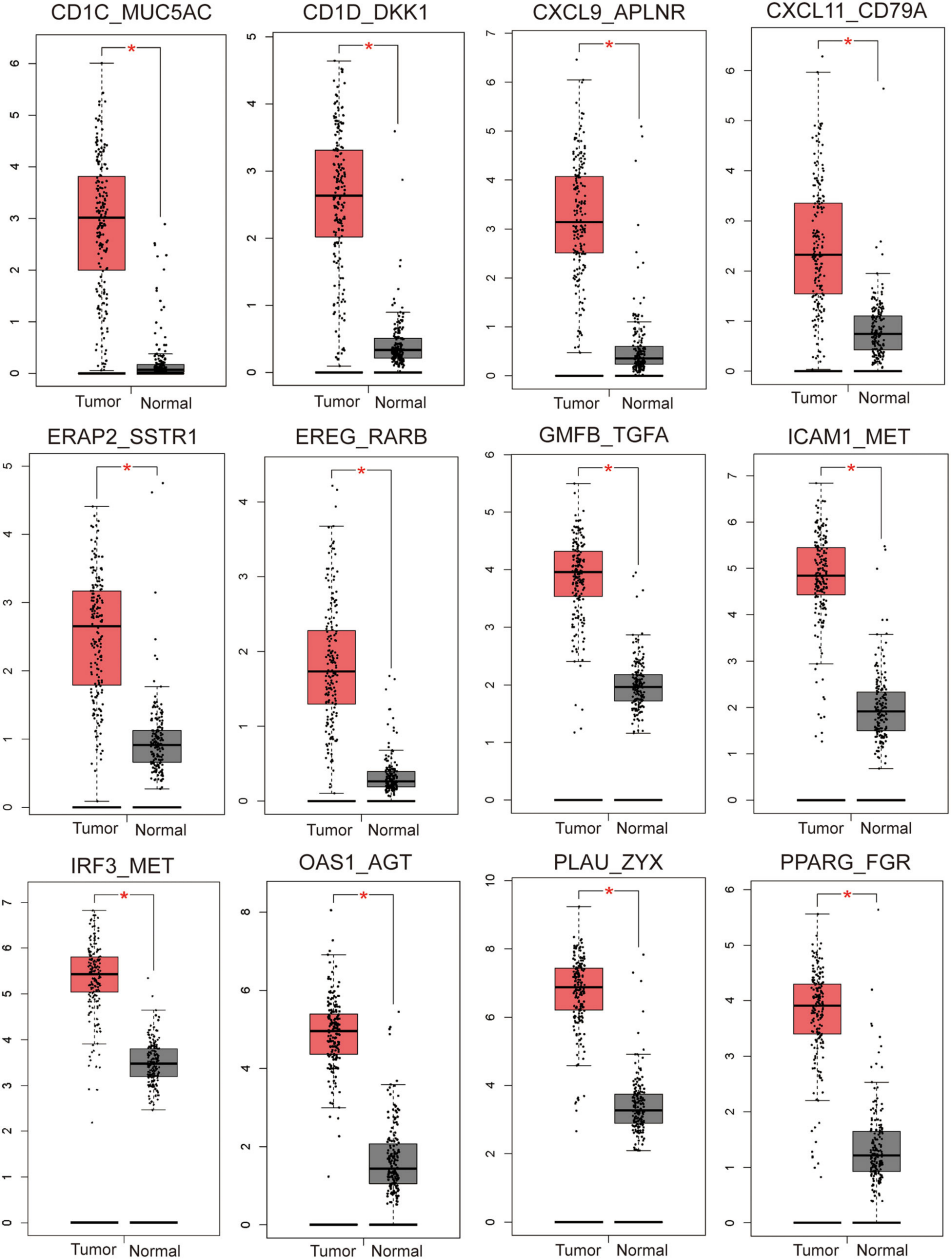
The mRNA expression of each IRGP in pancreatic cancer. Results revealed that CD1C_MUC5AC, CD1D_DKK1, CXCL9_APLNR, CXCL11_CD79A, ERAP2_SSTR1, EREG_RARB, GMFB_TGFA, ICAM1_MET, IRF3_MET, OAS1_AGT, PLAU_ZYX, and PPARG_FGR were overexpressed in pancreatic cancer. *p < .05. IRGP, immune‐related gene pair; mRNA, messenger RNA

**Figure 6:**
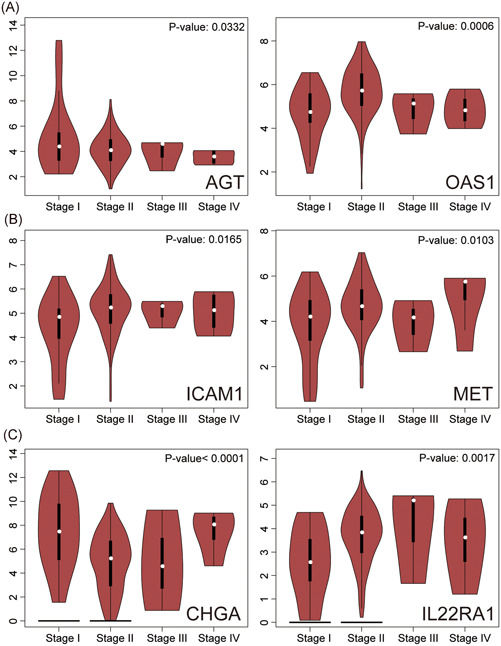
Correlation between the expression profile of each IRGP with pancreatic cancer stage. (A–C) Results revealed that mRNA expressions of AGT_OAS1, ICAM1_MET, and CHGA_IL22RA1 correlated with pancreatic cancer stage. IRGP, immune‐related gene pair; mRNA, messenger RNA

Results from GEPIA2 revealed that 12 IRGPs were upregulated in PC, which were CD1C_MUC5AC, CD1D_DKK1, CXCL9_APLNR, CXCL11_CD79A, ERAP2_SSTR1, EREG_RARB, GMFB_TGFA, ICAM1_MET, IRF3_MET, OAS1_AGT, PLAU_ZYX, and PPARG_FGR (Figure 5). Expression profile of AGT_OAS1, ICAM1_MET, and CHGA_IL22RA1 was correlated with PC stage (Figure 6).

### 3.8 Analysis of genetic alterations

**Figure 7:**
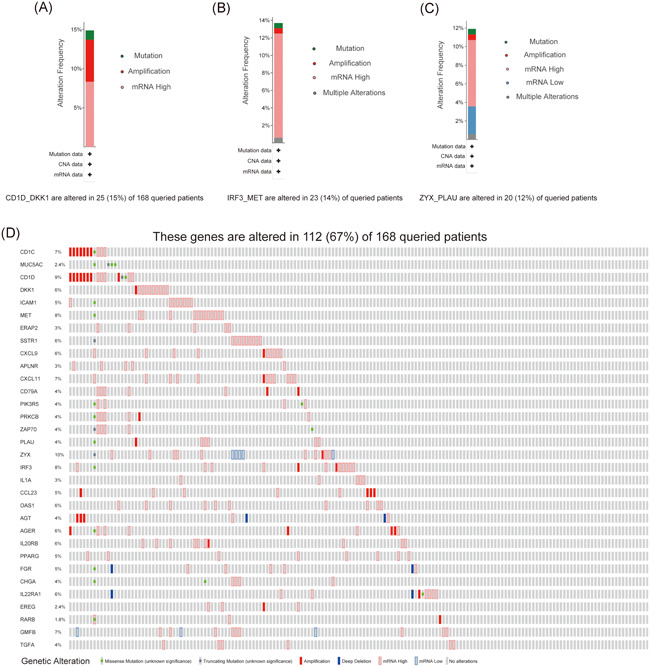
Mutation analysis of the IRGPs signature in pancreatic cancer. (A–C) The mutation rate of CD1D_DKK1 (15%), IRF3_MET (14%), and ZYX_PLAU (12%) was the top three highest in pancreatic cancer. The most common mutational category of the three IRGPs was mRNA high. Moreover, nine patients have CD1D_DKK1 genetic amplification, which is the highest among all IRGPs. (D) Results revealed that 112 (67%) of 168 pancreatic cancer samples have genetic alterations of the genes in the IRGPs signature, the most common mutational category was mRNA high. IRGP, immune‐related gene pair; mRNA, messenger RNA

Results from cBioportal revealed that 112 (67%) of the 168 PC patients have genetic alterations of at least one gene in the IRGPs signature and the most common genetic alteration category was messenger RNA (mRNA) high (Figure 7D). CD1D_DKK1 has the highest genetic alteration rate (15%), the most common genetic alteration category was mRNA high, besides, nine patients have CD1D_DKK1 genetic amplification, which is the highest among all IRGPs (Figure 7A). The mutation rate of IRF3_MET and ZYX_PLAU were 14% and 12%, respectively, which were the highest except CD1D_DKK1. The most common genetic alteration category of these two IRGPs was mRNA high (Figure 7B,C).

## 4 DISCUSSION

PC is one of the most lethal malignancies. Due to its components of plentiful desmoplastic stroma which could handicap the infiltration of effector T‐cell and further facilitate the immunosuppressive microenvironment, and the traits of poor immunogenicity, it was fairly intricate to accomplish immunotherapy into pancreatic cancer.^16^ Despite it was a gigantic challenge, previous literatures have implied the role of immune system in PC and supplied the potential feasibility to accomplish this target.^7^, ^8^, ^9^ As yet, the role of IRGs in PC is still unclear. In our study, we constructed an IRGPs signature to correlate with the prognosis of PC patients. Unlike the traditional prognostic model, the pairwise comparison of each IRGP and the calculation of the score were absolutely based on the genetic expressions in the same patient thus it is not a necessity in our IRGPs signature to standardize the genetic expression profiles from different sequencing platforms. Previous studies have demonstrated the validity of this method.^17^, ^18^

In our study, we construct an IRGPs signature to predict the prognosis of PC patients. The AUC of this model was 0.843, which demonstrated the validity of our prognostic signature. This signature contains 18 IRGPs, dividing all patients into high/low immune risk groups. Survival rate analysis and univariate/multivariate Cox proportional‐hazards analysis have not only demonstrated the prognostic value but also certified our IRGPs signature as an independent prognostic factor.

The contents of memory B cells, macrophages M0, macrophages M1 and NK cells activated were higher in the high‐risk group. The contents of naïve B cells and CD8 T cells were lower in the high‐risk group. Naïve B cells and CD8 T cells were demonstrated to be anticancer immune cells. Naïve B cells and CD8 T cells were gathered in CD31 high tumors, and the higher expression of CD31 implied more vascular endothelial cells, which was correlated with better prognosis in PC patients.^19^ Previous studies demonstrated that high density of tumor infiltrating naïve B cells is correlated with higher survival rates in hepatocellular carcinoma patients. It was also confirmed to be an independent prognostic factor in liver cancer. High density of naïve B cells was also validated to associate with tiny tumor size and good differentiation.^20^ Tumor‐associated macrophages in the tumor microenvironment usually promote cancer cell proliferation, immunosuppression and angiogenesis, thereby supporting tumor growth and metastasis. Moreover, the abundance of tumor‐associated macrophages was correlated with poor prognosis of patients.^21^ Macrophages contains different subtypes, including M0, M1, and M2. M0 macrophages is an inactive subtype which were not capable have inflammatory and tumor‐related functions. M1 macrophages and M2 macrophages were participated in diverse immune regulations and could derived from M0 macrophages.^22^ High contents of M0 macrophages was demonstrated to correlate with poor prognosis of PC patients.^23^ The contents of M0 and M1 macrophages were higher in colorectal cancer tissues than paired normal tissues. Moreover, abundance of M1 macrophages was correlated with poor prognosis of colorectal cancer patients.^24^ Coculture of macrophages and PC cells in vitro remarkably enhanced the expression of CD163 and programmed death‐ligand 1 (PD‐L1), which were two risk factors and correlated with poor prognosis of PC patients.^25^ Memory B cells are produced in the germinal center response during the T‐cell‐dependent immune response. Several B‐cell malignancies including chronic lymphocytic leukemia, hairy cell leukemia and marginal zone lymphomas were demonstrated to derived from memory B cells. The activation of memory B cells correlated with the progression of these malignancies.^26^

The results of GSEA revealed our IRGPs signature mainly involved in ion channel activity and gated channel activity, especially in potassium and voltage gating channels. Ion channels have been shown to play an important role in the occurrence and development of diverse cancers. Potassium channels including four categories, which are voltage gate (Kv), calcium dependent (KCa), two‐hole domain group (K2p) and inward rectification (Kir).^27^ KCa3.1 is a K^+^ channel activated by Ca^2+^, which was overexpressed in PC and correlated with poor prognosis. It was demonstrated to functioned in the progression of many cancers and is related to the migration and proliferation of PC cells.^28^ Kv11.1 was demonstrated to be overexpressed in PC, overexpression of Kv11.1 was correlated with poor differentiation and larger tumor size.^29^

Results revealed that 12 IRGPs were overexpressed in PC. Expressions of AGT_OAS1, IACM1_MET, and CHGA_IL22RA1 were associated with PC stage. Mutational analysis revealed that the genetic alteration rate of CD1D_DKK1, IRF3_MET, ZYX_PLAU were high in PC and the most common alteration category were mRNA high. Above results illustrated the correlation between our model with PC. Moreover, KEGG analysis revealed that our IRGPs signature was correlated with cancer pathways. Previous literatures also demonstrated the correlation between our IRGPs signature with the development of cancers including PC. DKK1 is a secreted protein that prohibited the β‐catenin‐dependent pathway in Wnt signaling by binding to Wnt receptors. Dysregulation of DKK1 was reported to correlated with the prognosis and progression of various cancers including PC.^30^, ^31^, ^32^ DKK1 was overexpressed in PC and Dkk1‐CKAP4‐PI3K/AKT signaling pathway affected PC cells proliferation.^32^ IL22RA1 is a member of the class II cytokine receptor family. Previous study uncovered IL22RA1 was overexpressed in PC and the expression was correlated with poor prognosis. IL22RA1/STAT3 signaling promoted stemness and tumorigenicity in PC.^33^ ICAM1 has been reported to associated with cancer metastasis, including PC. The activation of IACAM1 is activated by interleukin‐35 through the GP130‐STAT1 signaling pathway.^34^ PLAU is a urokinase plasminogen activator which could promote the proteolytic cascade. It has been demonstrated to associated with the invasion and metastasis of cancers.^35^, ^36^ Upregulation of PLAU was correlated with lymph node metastasis and poor prognosis of PC. Low‐expression of PLAU prohibited proliferation and migration of PC cells.^37^ Moreover, the expression of CD1D, ERAP2, SSTR1, CXCL9, CXCL11, IL1A, EREG also certainly influenced the development of PC.^38^, ^39^, ^40^, ^41^, ^42^, ^43^, ^44^, ^45^, ^46^ Hence, these studies demonstrated the correlation between our IRGPs signature with the progression of PC.

The aim of this study was to apply IRGs into the treatment of PC. Despite we have demonstrated the validity of our IRGPs signature and verified the correlation between our model and immune cells, we also acknowledge the limitation in our study. We expect further experiments including western blot or immunohistochemistry could be performed in our study.

## 5 CONCLUSION

In conclusion, our IRGPs signature could predict the prognosis of PC patients, and this prognostic signature will facilitate the application of immunotherapy in PC.

## Data Availability

The data that support the findings of this study are openly available in TCGA database and GSE57495 data set in GEO database.

## References

[1] Kamisawa T , Wood LD , Itoi T , Takaori K . Pancreatic cancer. Lancet. 2016;388(10039):73‐85. 26830752 10.1016/S0140-6736(16)00141-0

[2] Rawla P , Sunkara T , Gaduputi V . Epidemiology of pancreatic cancer: global trends, etiology and risk factors. World J Oncol. 2019;10(1):10‐27. 30834048 10.14740/wjon1166 PMC6396775

[3] Zhu H , Li T , Du Y , Li M . Pancreatic cancer: challenges and opportunities. BMC Med. 2018;16(1):214. 30463539 10.1186/s12916-018-1215-3 PMC6249728

[4] Gentles AJ , Newman AM , Liu CL , et al. The prognostic landscape of genes and infiltrating immune cells across human cancers. Nat Med. 2015;21(8):938‐945. 26193342 10.1038/nm.3909 PMC4852857

[5] Aravalli RN . Role of innate immunity in the development of hepatocellular carcinoma. World J Gastroenterol. 2013;19(43):7500‐7514. 24282342 10.3748/wjg.v19.i43.7500 PMC3837249

[6] Ganesh K , Stadler ZK , Cercek A , et al. Immunotherapy in colorectal cancer: rationale, challenges and potential. Nat Rev Gastroenterol Hepatol. 2019;16:361‐375. 30886395 10.1038/s41575-019-0126-x PMC7295073

[7] Basso D , Fogar P , Falconi M , et al. Pancreatic tumors and immature immunosuppressive myeloid cells in blood and spleen: role of inhibitory co‐stimulatory molecules PDL1 and CTLA4. An in vivo and in vitro study. PLOS One. 2013;8(1):e54824. 23359812 10.1371/journal.pone.0054824 PMC3554636

[8] Ino Y , Yamazaki‐Itoh R , Shimada K , et al. Immune cell infiltration as an indicator of the immune microenvironment of pancreatic cancer. Br J Cancer. 2013;108(4):914‐923. 23385730 10.1038/bjc.2013.32 PMC3590668

[9] Suzuki D , Furukawa K , Kimura F , et al. Effects of perioperative immunonutrition on cell‐mediated immunity, T helper type 1 (Th1)/Th2 differentiation, and Th17 response after pancreaticoduodenectomy. Surgery. 2010;148(3):573‐581. 20227099 10.1016/j.surg.2010.01.017

[10] Peng YP , Xi CH , Zhu Y , et al. Altered expression of CD226 and CD96 on natural killer cells in patients with pancreatic cancer. Oncotarget. 2016;7(41):66586‐66594. 27626490 10.18632/oncotarget.11953 PMC5341822

[11] Bhattacharya S , Dunn P , Thomas CG , et al. ImmPort, toward repurposing of open access immunological assay data for translational and clinical research. Sci Data. 2018;5:180015. 29485622 10.1038/sdata.2018.15 PMC5827693

[12] Newman AM , Liu CL , Green MR , et al. Robust enumeration of cell subsets from tissue expression profiles. Nat Methods. 2015;12(5):453‐457. 25822800 10.1038/nmeth.3337 PMC4739640

[13] Huang DW , Sherman BT , Tan Q , et al. DAVID bioinformatics resources: expanded annotation database and novel algorithms to better extract biology from large gene lists. Nucleic Acids Res. 2007;35:W169‐W175. 17576678 10.1093/nar/gkm415 PMC1933169

[14] Tang Z , Kang B , Li C , Chen T , Zhang Z . GEPIA2: an enhanced web server for large‐scale expression profiling and interactive analysis. Nucleic Acids Res. 2019;47(W1):W556‐W560. 31114875 10.1093/nar/gkz430 PMC6602440

[15] Jiao XD , Qin BD , You P , Cai J , Zang YS . The prognostic value of TP53 and its correlation with EGFR mutation in advanced non‐small cell lung cancer, an analysis based on cBioPortal data base. Lung Cancer. 2018;123:70‐75. 30089598 10.1016/j.lungcan.2018.07.003

[16] Torphy RJ , Schulick RD , Zhu Y . Understanding the immune landscape and tumor microenvironment of pancreatic cancer to improve immunotherapy. Mol Carcinog. 2020;59:775‐782. 10.1002/mc.23179 32166821

[17] Popovici V , Budinska E , Tejpar S , et al. Identification of a poor‐prognosis BRAF‐mutant‐like population of patients with colon cancer. J Clin Oncol. 2012;30(12):1288‐1295. 22393095 10.1200/JCO.2011.39.5814

[18] Peng PL , Zhou XY , Yi GD , Chen PF , Wang F , Dong WG . Identification of a novel gene pairs signature in the prognosis of gastric cancer. Cancer Med. 2018;7(2):344‐350. 29282891 10.1002/cam4.1303 PMC5806102

[19] Katsuta E , Qi Q , Peng X , Hochwald SN , Yan L , Takabe K . Pancreatic adenocarcinomas with mature blood vessels have better overall survival. Sci Rep. 2019;9(1):1310. 30718678 10.1038/s41598-018-37909-5 PMC6362082

[20] Zhang Z , Ma L , Goswami S , et al. Landscape of infiltrating B cells and their clinical significance in human hepatocellular carcinoma. Oncoimmunology. 2019;8(4):e1571388. 30906667 10.1080/2162402X.2019.1571388 PMC6422393

[21] Ngambenjawong C , Gustafson HH , Pun SH . Progress in tumor‐associated macrophage (TAM)‐targeted therapeutics. Adv Drug Deliv Rev. 2017;114:206‐221. 28449873 10.1016/j.addr.2017.04.010 PMC5581987

[22] Hume DA . The many alternative faces of macrophage activation. Front Immunol. 2015;6:370. 26257737 10.3389/fimmu.2015.00370 PMC4510422

[23] Xu C , Sui S , Shang Y , et al. The landscape of immune cell infiltration and its clinical implications of pancreatic ductal adenocarcinoma. J Adv Res. 2020;24:139‐148. 32322419 10.1016/j.jare.2020.03.009 PMC7171261

[24] Ge P , Wang W , Li L , et al. Profiles of immune cell infiltration and immune‐related genes in the tumor microenvironment of colorectal cancer. Biomed Pharmacother. 2019;118:109228. 31351430 10.1016/j.biopha.2019.109228

[25] Xu JY , Wang WS , Zhou J , et al. The importance of a conjoint analysis of tumor‐associated macrophages and immune checkpoints in pancreatic cancer. Pancreas. 2019;48(7):904‐912. 31268976 10.1097/MPA.0000000000001364

[26] Seifert M , Küppers R . Human memory B cells. Leukemia. 2016;30(12):2283‐2292. 27499139 10.1038/leu.2016.226

[27] Felipe A , Vicente R , Villalonga N , et al. Potassium channels: new targets in cancer therapy. Cancer Detect Prev. 2006;30(4):375‐385. 16971052 10.1016/j.cdp.2006.06.002

[28] Bonito B , Sauter DRP , Schwab A , Djamgoz MBA , Novak I . KCa3.1 (IK) modulates pancreatic cancer cell migration, invasion and proliferation: anomalous effects on TRAM‐34. Pflugers Arch. 2016;468(11–12):1865‐1875. 27752766 10.1007/s00424-016-1891-9

[29] Feng J , Yu J , Pan X , et al. HERG1 functions as an oncogene in pancreatic cancer and is downregulated by miR‐96. Oncotarget. 2014;5(14):5832‐5844. 25071021 10.18632/oncotarget.2200 PMC4170607

[30] Sun W , Shang J , Zhang J , et al. Correlations of DKK1 with incidence and prognosis of breast cancer. J BUON. 2019;24(1):26‐32. 30941948

[31] Zhuang X , Zhang H , Li X , et al. Differential effects on lung and bone metastasis of breast cancer by Wnt signalling inhibitor DKK1. Nat Cell Biol. 2017;19(10):1274‐1285. 28892080 10.1038/ncb3613

[32] Igbinigie E , Guo F , Jiang SW , Kelley C , Li J . Dkk1 involvement and its potential as a biomarker in pancreatic ductal adenocarcinoma. Clin Chim Acta. 2019;488:226‐234. 30452897 10.1016/j.cca.2018.11.023

[33] He W , Wu J , Shi J , et al. IL22RA1/STAT3 signaling promotes stemness and tumorigenicity in pancreatic cancer. Cancer Res. 2018;78(12):3293‐3305. 29572224 10.1158/0008-5472.CAN-17-3131

[34] Huang C , Li N , Li Z , et al. Tumour‐derived Interleukin 35 promotes pancreatic ductal adenocarcinoma cell extravasation and metastasis by inducing ICAM1 expression. Nat Commun. 2017;8:14035. 28102193 10.1038/ncomms14035 PMC5253665

[35] Mauro CD , Pesapane A , Formisano L , et al. Urokinase‐type plasminogen activator receptor (uPAR) expression enhances invasion and metastasis in RAS mutated tumors. Sci Rep. 2017;7(1):9388. 28839232 10.1038/s41598-017-10062-1 PMC5571185

[36] Moirangthem A , Bondhopadhyay B , Mukherjee M , et al. Simultaneous knockdown of uPA and MMP9 can reduce breast cancer progression by increasing cell‐cell adhesion and modulating EMT genes. Sci Rep. 2016;6:21903. 26906973 10.1038/srep21903 PMC4764826

[37] Zhao X , Liu Z , Ren Z , et al. Triptolide inhibits pancreatic cancer cell proliferation and migration via down‐regulating PLAU based on network pharmacology of *Tripterygium wilfordii* Hook F. Eur J Pharmacol. 2020;880:173225. 32464191 10.1016/j.ejphar.2020.173225

[38] Das S , Bar‐Sagi D . BTK signaling drives CD1dhiCD5+ regulatory B‐cell differentiation to promote pancreatic carcinogenesis. Oncogene. 2019;38(17):3316‐3324. 30635655 10.1038/s41388-018-0668-3 PMC6486434

[39] Wu G , Deng Z , Jin Z , et al. Identification of prognostic immune‐related genes in pancreatic adenocarcinoma and establishment of a prognostic nomogram: a bioinformatic study. BioMed Res Int. 2020;2020:1346045‐15. 32596278 10.1155/2020/1346045 PMC7301181

[40] Li M , Wang X , Li W , et al. Somatostatin receptor‐1 induces cell cycle arrest and inhibits tumor growth in pancreatic cancer. Cancer Sci. 2008;99(11):2218‐2223. 18823376 10.1111/j.1349-7006.2008.00940.x PMC2930023

[41] Li M , Fisher WE , Kim HJ , et al. Somatostatin, somatostatin receptors, and pancreatic cancer. World J Surg. 2005;29(3):293‐296. 15706439 10.1007/s00268-004-7814-5

[42] Qian L , Yu S , Yin C , et al. Plasma IFN‐γ‐inducible chemokines CXCL9 and CXCL10 correlate with survival and chemotherapeutic efficacy in advanced pancreatic ductal adenocarcinoma. Pancreatology. 2019;19(2):340‐345. 30685120 10.1016/j.pan.2019.01.015

[43] Gao HF , Cheng CS , Tang J , et al. CXCL9 chemokine promotes the progression of human pancreatic adenocarcinoma through STAT3‐dependent cytotoxic T lymphocyte suppression. Aging. 2020;12(1):502‐517. 31913856 10.18632/aging.102638 PMC6977695

[44] Ge WL , Chen Q , Meng LD , et al. The YY1/miR‐548t‐5p/CXCL11 signaling axis regulates cell proliferation and metastasis in human pancreatic cancer. Cell Death Dis. 2020;11(4):294. 32341359 10.1038/s41419-020-2475-3 PMC7186231

[45] Brunetto E , De Monte L , Balzano G , et al. The IL‐1/IL‐1 receptor axis and tumor cell released inflammasome adaptor ASC are key regulators of TSLP secretion by cancer associated fibroblasts in pancreatic cancer. J Immunother Cancer. 2019;7(1):45. 30760333 10.1186/s40425-019-0521-4 PMC6373075

[46] Carpenter BL , Chen M , Knifley T , et al. Integrin α6β4 promotes autocrine epidermal growth factor receptor (EGFR) signaling to stimulate migration and invasion toward hepatocyte growth factor (HGF). J Biol Chem. 2015;290(45):27228‐27238. 26381405 10.1074/jbc.M115.686873 PMC4646402

